# Habitat restoration weakens negative environmental effects on telomere dynamics

**DOI:** 10.1111/mec.15980

**Published:** 2021-07-22

**Authors:** Darryl McLennan, Sonya K. Auer, Simon McKelvey, Lynn McKelvey, Graeme Anderson, Winnie Boner, Jessica S. Duprez, Neil B. Metcalfe

**Affiliations:** ^1^ Institute of Biodiversity, Animal Health and Comparative Medicine University of Glasgow Glasgow UK; ^2^ Department of Biology Williams College Williamstown MA USA; ^3^ Cromarty Firth Fishery Trust Inverness UK

**Keywords:** conservation biology, environmental variation, food availability, *Salmo*, senescence, telomerase

## Abstract

Habitat quality can have far‐reaching effects on organismal fitness, an issue of concern given the current scale of habitat degradation. Many temperate upland streams have reduced nutrient levels due to human activity. Nutrient restoration confers benefits in terms of invertebrate food availability and subsequent fish growth rates. Here we test whether these mitigation measures also affect the rate of cellular ageing of the fish, measured in terms of the telomeres that cap the ends of eukaryotic chromosomes. We equally distributed Atlantic salmon eggs from the same 30 focal families into 10 human‐impacted oligotrophic streams in northern Scotland. Nutrient levels in five of the streams were restored by simulating the deposition of a small number of adult Atlantic salmon *Salmo salar* carcasses at the end of the spawning period, while five reference streams were left as controls. Telomere lengths and expression of the telomerase reverse transcriptase (TERT) gene that may act to lengthen telomeres were then measured in the young fish when 15 months old. While TERT expression was unrelated to any of the measured variables, telomere lengths were shorter in salmon living at higher densities and in areas with a lower availability of the preferred substrate (cobbles and boulders). However, the adverse effects of these habitat features were much reduced in the streams receiving nutrients. These results suggest that adverse environmental pressures are weakened when nutrients are restored, presumably because the resulting increase in food supply reduces levels of both competition and stress.

## 1 INTRODUCTION

Habitats range in quality from more optimal “high‐quality” habitats that suitably meet the ecological requirements of the animals that live within them to suboptimal “lower quality” habitats that fail to meet one or more of these requirements. While environmental heterogeneity (and therefore variation in habitat quality) exists naturally, habitats are increasingly being degraded due to human activity. As a consequence, the quality of a given habitat may be impacted by factors such as pollution (Cooper, 1993; Nedeau et al., 2003; Sanderfoot & Holloway, 2017), disturbance and loss of connectivity (Hodgson et al., 2011; Magris et al., 2016; Stevens & Baguette, 2008; Tripp et al., 2019) as well as the alteration of food webs and associated prey availability (Hempson et al., 2017; Murphy et al., 2019; Paviolo et al., 2018; Saporiti et al., 2014). However, with a shift towards better conservation management in recent times, there has been a greater focus on how detrimental anthropogenic effects may be better mitigated and how the quality of impacted habitats may be restored (Miller & Hobbs, 2007; Newmark et al., 2017; Tonietto & Larkin, 2018).

The condition of a given habitat can carry costs for the long‐term fitness of the organisms that live within it (Cīrule et al., 2017; Josserand et al., 2017; Lea et al., 2018; Pironon et al., 2017). Endeavours to measure the effects of habitat conditions on individual performance could benefit from measuring the length of the telomeres: nucleoprotein structures that cap the ends of eukaryotic chromosomes (Blackburn, 1991; Monaghan, 2010). These telomere caps play a fundamental role in allowing cells to distinguish between natural chromosome ends and double stranded DNA breaks, thus preventing chromosomal fusion. Importantly, they also help buffer the coding region of a chromosome from the “end replication problem,” whereby chromosomes are not fully able to replicate, and so a number of base pairs are lost from the chromosome ends with each cell division (Shay & Wright, 2019). Due to the terminal location of the telomere caps, it is the noncoding telomeric sequence that is lost during this process, thereby protecting the central coding DNA. While telomere elongation mechanisms such as the expression of the enzyme telomerase may act to counterbalance this loss, such mechanisms are often downregulated in post‐embryonic somatic tissues (Gomes et al., 2010; Tian et al., 2018). Therefore, in the absence of elongation mechanisms, telomeres may shorten to such an extent that the central coding region of a chromosome becomes vulnerable, which can then trigger the senescence or death of that cell (Victorelli & Passos, 2017; Zhu et al., 2019). This considered, a relatively short telomere length is thought to be an indicator of poor cellular and biological state, and a number of studies have linked a shorter telomere length and/or a faster rate of telomere attrition to reduced survival and/or longevity (Boonekamp et al., 2014; Noguera et al., 2018; Olsson et al., 2018; Wilbourn et al., 2018; Wood & Young, 2019).

The observed rate of telomere loss is often much greater than if it were occurring solely because of cell division, leading to the hypothesis that telomere shortening is also under the influence of environmental stressors (Angelier et al., 2018; Chatelain et al., 2020). Though not yet fully understood, the most predominantly suggested link between environmental conditions and telomere dynamics are oxidative stress pathways (Barnes et al., 2019; Coluzzi et al., 2019; Reichert & Stier, 2017). This is partly because exposure to nonoptimal environmental conditions can trigger complex physiological stress responses (via the hypothalamic–pituitary–adrenal axis) that are associated with elevated rates of aerobic metabolism and ATP production, which in turn can increase the production of reactive oxygen species (ROS) as by‐product. If left unquenched, ROS may cause oxidative damage to cellular molecules such as lipids, proteins and DNA. Telomeric DNA has a high guanine content, which is known to be particularly susceptible to oxidative damage and is hard to repair in the telomeric regions (Kawanishi & Oikawa, 2004; Monaghan & Ozanne, 2018; Passos et al., 2007).

Rates of telomere attrition have been linked to abiotic environmental factors such as temperature (Axelsson et al., 2020; Debes et al., 2016; Dupoué et al., 2017; Foley et al., 2020), and to biotic factors such as social position and population density (Kotrschal et al., 2007; McLennan et al., 2016; Nettle et al., 2015; Sohn et al., 2012). Therefore, if a given population is spread between habitats of contrasting quality (caused by marked differences in one or more environmental factors and/or the interaction between these), this may also result in differences in average telomere length between the contrasting habitats. A handful of studies have investigated populations split between habitats of contrasting quality, with the general finding that habitats of poorer quality carry costs (in terms of faster telomere attrition) for the individuals that live within them. Examples of contrasting habitats that have been studied thus far include low vs. high elevation (McLennan et al., 2016), low vs. high food availability (Angelier et al., 2013), and urban vs. rural environments (Grunst et al., 2019; Meillère et al., 2015; Salmón et al., 2016). However, to our knowledge none of these studies have investigated how the restoration of lower quality habitats might help to mitigate these potentially negative effects on the telomere dynamics of the animals that live within them.

In this study, we examined whether habitat quality impacts the telomere dynamics of juvenile Atlantic salmon *Salmo salar* living in Scottish upland streams. More specifically, we tested whether habitat restoration of human‐impacted oligotrophic streams can have direct and/or interactive effects on a salmon's telomere length and rate of telomerase expression (measured here by quantifying telomerase reverse transcriptase [TERT] expression). Atlantic salmon are an anadromous species in which individuals are born in fresh water, migrate to the ocean to achieve a greater growth potential, and then return from the ocean to reproduce in their natal river or stream. Scottish populations (such as the one used in this study) are generally either 2 or 3 years old at the time of seaward migration, and then spend either one or two winters at sea prior to their return migration (McLennan et al., 2017). These returning individuals act as important vectors for marine‐derived nutrients, via the production of waste products, gametes and the decomposition of carcasses that arise from post‐spawning mortality (Nislow et al., 2004; Samways & Cunjak, 2015; Willson & Halupka, 1995). The small upland streams in which salmon often spawn are typically nutrient‐poor (Elliott et al., 1998; Nislow et al., 2004), and so depend heavily on these annual nutrient pulses to maintain the architecture of the food webs within them and thus benefit the offspring that arise from the salmon spawnings (McLennan et al., 2019; Nislow et al., 2010; Samways et al., 2018). However, there have been recent declines in adult populations, partly due to a rise in the number of hydraulic structures (often for the purpose of hydropower) that can act as barriers to migration (Lenders et al., 2016; Limburg & Waldman, 2009). This has led to reduced nutrient subsidies to the habitats upriver of these barriers, which in turn has reduced the productivity of these once abundant salmon spawning grounds (Williams et al., 2009). It is possible to restore nutrient levels in these freshwater habitats to some presumed previous level, such as via the addition of fish carcasses or fish carcass “analogues” (Bilby et al., 1998; Guyette et al., 2013; Kohler et al., 2012; Williams et al., 2009). Previous studies have shown that when carefully managed and evidence‐based, nutrient restoration can have substantial and sustained positive effects on the prey availability, growth rates and genetic diversity of juvenile salmon (Auer et al., 2020; McLennan et al., 2019; Williams et al., 2009; Wipfli et al., 2003). This has led to the consensus that nutrient restoration acts to increase the relative quality of these juvenile salmon habitats. However, the effects of restoration on the longer term physiological fitness of the juvenile salmon remain little tested. Moreover, how this process of nutrient restoration may interact to strengthen or weaken other potential environmental effects has not yet been examined.

We have previously shown, both in the laboratory and in the field, that the telomere dynamics of juvenile Atlantic salmon are under the influence of multiple environmental factors, such as temperature and population density (McLennan et al., 2016; McLennan et al., 2018a). For this study, we adopted an experimental approach in the field, whereby Atlantic salmon siblings (of known family origin) were reared in contrasting habitats: either in “high‐quality” streams that underwent nutrient restoration or in “low‐quality” streams that remained as nutrient‐poor controls. Alongside this, we also simultaneously quantified (both within and among streams) four of the most important habitat variables for juvenile salmon: local juvenile salmon density, stream depth, water velocity and substrate structure (Aas et al., 2011; Jonsson & Jonsson, 2011). By adopting this approach, we not only tested whether this habitat restoration (in the form of nutrient subsidies) can have direct effects on the telomere dynamics of the juvenile salmon, but also whether this increase in habitat quality may act to strengthen or weaken the potential effects that the other four measured habitat variables have on the telomere dynamics of the juvenile salmon.

## 2 MATERIALS AND METHODS

### 2.1 Focal salmon families

We used in vitro fertilization to create 30 focal salmon families over a 3‐day period in December 2015, at the River Conon catchment in northern Scotland. Wild parental fish were captured at the Loch na Croic fish trap (57°60′N, 4°63′W) during their return spawning migration from the sea. Thirty randomly chosen females that had spent a single winter at sea (confirmed by scale reading; Shearer, 1992) were crossed with 30 randomly chosen males. Prior to the stripping of gametes, parents were anaesthetized using a mild 5% benzocaine solution. Each female was stripped of eggs that were then fertilized with sperm from one of the males to create full‐sibling families. A small sample of adipose fin tissue was collected from each parent for genotyping (to allow subsequent parental assignment of the resulting offspring). Each parent was measured (fork length to 0.5 cm; body mass to 0.1 g) and then returned to the river. The 30 families of eggs were then kept over winter at the nearby Contin hatchery, where they were reared in family‐specific trays.

### 2.2 Field protocol

We selected 10 small headwater tributaries within the River Conon catchment to be used as study streams (Figure S1). These streams provide suitable habitat for juvenile salmon but support no natural spawning due to hydropower dams within the catchment preventing parent fish from migrating into the streams. As a result, none of these streams receive a natural net‐import of marine‐derived nutrients, thereby limiting their potential productivity (McLennan et al., 2019; Williams et al., 2009). In late February to early March 2016, the developing eggs from the hatchery were planted out into the 10 study streams. Each stream received 3000 eggs, with 100 eggs coming from each of the 30 focal salmon families (mixed together). In each of the streams, eggs were planted out in a 300‐m^2^ experimental reach that was 75–100 m long, depending on stream width. Two Vibert boxes containing 100 eggs each were placed at the upper and lower limits of each experimental reach (one box at each limit) in order to assess hatching success. The Vibert boxes were found to be empty when retrieved in late May to early June 2016, indicating successful hatching in all streams. The remaining 2800 eggs were planted out in eight equal‐sized artificial nests (similar to McLennan et al., 2016), each containing a mix of all families and spread between the upper and lower Vibert boxes.

### 2.3 Nutrient restoration

Five of the study streams were randomly assigned to undergo nutrient restoration (hereafter referred to as the “high nutrient” treatment) and the other five study streams were left as nutrient‐poor controls (hereafter referred to as the “low nutrient” treatment). While no manipulation occurred in the low nutrient streams, the high nutrient streams received analogue carcasses composed of fish feed pellets (Coral 2000+40PAX B12, Skretting) that contained 60% marine‐derived fish‐based nutrients and were similar to salmon carcasses in their nutritional content and decay rate (Pearsons et al., 2007). Each of the high nutrient streams received 15 kg of pellets that were divided among five 3‐kg mesh bags. The mesh bags were evenly spaced along the experimental reach and were anchored to the stream bed with large rocks to prevent removal of the pellets by scavengers. This 15‐kg nutrient treatment is thought to be the equivalent of 25 adult salmon carcasses; an amount similar to or less than that used in other nutrient restoration experiments in Atlantic salmon streams (Guyette et al., 2013, 2014; Williams et al., 2009). The five high nutrient streams received this nutrient treatment on two occasions: first in March 2016 (at the time of egg planting) and then again 1 year later in March 2017 to simulate the yearly deposition of adult carcasses. We have previously shown that this treatment resulted in an improvement in habitat quality, with the high nutrient streams having consistently higher levels of macroinvertebrate abundance and biomass throughout the experimental period (McLennan et al., 2019). At the time of the second nutrient addition, a second cohort of (nonfocal) salmon eggs was added at the same density as before in order to maintain a normal age structure of juvenile salmon in the 10 study streams. Temporal changes in daily water temperature were monitored in eight of the study streams (four low nutrient and four high nutrient) from the time that the focal eggs were planted out to the recapture of the focal juvenile salmon at age 1+ years old (March 2016 to July 2017), using HOBO temperature data loggers (Onset Computer Corporation) that were programmed to record data every 4 h (Figure S2).

### 2.4 Recapture of the focal juvenile salmon

The resulting offspring of the 30 focal families were in their second summer of life (aged 1+ years old) when each of the streams was sampled between July 12 and 22, 2017. At this time, experimental reaches were divided into subsections marked out every 5 m along the length of the surveyed reach, resulting in 16–20 subsections per stream, depending on the length of the experimental reach. Triple‐pass electrofishing was conducted throughout the experimental reaches, and the subsection in which each focal salmon was captured (i.e., its capture location) was recorded. When combined with measurements of the stream width of each subsection, this allowed the calculation of subsection‐specific 1+ salmon densities (no. of focal individuals/m^2^); this degree of spatial resolution is appropriate because previous studies have found that the growth of juvenile salmon varies with conspecific density measured over a similar sized area (Einum et al., 2011). Each focal salmon was anaesthetized (with clove oil 20 ppm) and measured for body mass (±1.0 mg) and body fork length (±0.01 mm). Focal salmon (i.e., those that were planted out in March 2016 and were 1+ years old at the time of recapture) were easily distinguishable from nonfocal salmon (i.e., those that were a year younger, having been planted out as eggs the following year in March 2017) due to the distinct difference in body size between the two cohorts; size differences between focal and nonfocal cohorts were confirmed by anaesthetizing and measuring 20 randomly selected nonfocal individuals per stream (Figure S3). A small sample of adipose fin tissue was taken from each focal individual and stored in 100% ethanol for subsequent DNA analyses (relative telomere length, assignment of sex and genotyping for parental assignment). At each stream, we also randomly selected 20 focal individuals to be euthanized so that a sample of anterior white muscle could be taken and stored in Allprotect reagent (Qiagen) for subsequent RNA extraction and the measurement of relative TERT expression (Srinivas et al., 2020). These randomly selected individuals were representative of the entire experimental reach of each stream, with a mean of 1.86 focal individuals (±SD 1.04) being sampled per subsection. Each recaptured focal salmon was genotyped and assigned to one of the 30 focal families by commercial suppliers (Landcatch Natural Selection Ltd). The same commercial supplier also assigned sex to each of these salmon; see Appendix S1 for details of the parental assignment and sex assignment protocols. In total, 90% of the focal juvenile salmon that had been recaptured within the experimental subsections were successfully assigned to one of the 30 focal families and were subsequently used in this study for relative telomere length measurement (*n* = 433) and relative TERT expression measurement (*n* = 175). The other 10% (those not successfully assigned to the focal families) were probably 1+ year old immigrating fish that had been stocked out into nearby nonexperimental streams in spring 2016, as part of routine fisheries management practice. While these immigrating fish were not used for subsequent molecular analyses, they were still included in the calculation of subsection‐specific densities. Of the 433 captured and assigned juvenile salmon, 226 were from the low nutrient streams (i.e., a recapture rate of 1.5%) and 207 were from the high nutrient streams (a recapture rate of 1.4%).

### 2.5 Habitat surveys

On the same day of the fish sampling, the microhabitat within each 5‐m subsection of our 10 study streams was characterized following the Scottish Fisheries Coordination Centre habitat survey protocol (SFCC, 2007). The same two observers made these assessments across all streams, after initial training and standardization. In addition to quantifying density, abiotic microhabitat variables including water depth, water flow and substrate size were also measured because these are thought to be three of the most important determinants of juvenile salmon microhabitat quality (Hedger et al., 2005; Heggenes et al., 1999; Jonsson & Jonsson, 2011). The mean water depth (cm) of each 5‐m subsection was based on 10 depth measurements (five equidistant points across the width of the stream at the upstream and downstream limits of each subsection). Water velocity was categorized into six flow classes, following the SFCC (2007) categories: Class 1 = still marginal (water still or eddying and silent), 2 = pool (water flow slow, eddying and silent), 3 = glide (water flow moderate to fast but silent and unbroken), 4 = run (water flow fast, unbroken standing waves at surface, silent), 5 = riffle (water flow fast, broken standing waves at surface, audible) and 6 = torrent (white water, noisy, substrate not visible). The proportion of each flow class within a subsection was estimated to the nearest 5% through visual assessment, and combined to calculate a flow index as *F*_i_ = Σ(*F*_c_ *F*_u_), where *F*_i_ is the flow index for each subsection, *F*_c_ is the flow class (1–6) and *F*_u_ is the proportion of the section composed of that class (Hedger et al., 2005; Rollinson & Hutchings, 2013). The flow index thus ranged from 1 to 6, with higher values indicating faster flowing water. Substrate size was assessed using a granulometric index based on a similar calculation (Hedger et al., 2005). Substrate was categorized into six classes according to particle size, following SFCC (2007): Class 1 = sand (≤2 mm), 2 = gravel (2–16 mm), 3 = pebble (16–64 mm), 4 = cobble (64–256 mm), 5 = boulder (>256 mm) and 6 = bedrock (continuous rock surface). The proportion of each particle size class was estimated to the nearest 5% through visual assessment and combined to calculate a granulometric index for each subsection as *G*_i_ = Σ(*G*_c_ *G*_u_), where *G*_i_ is the granulometric index for each subsection, *G*_c_ is the particle size class (1–6) and *G*_u_ is the proportion of the section composed by that class (Hedger et al., 2005). The granulometric index also ranged from 1 to 6 with higher values indicating a predominance of larger substrate sizes. The four measured habitat variables (density, flow index, depth and granulometric index) did not differ significantly between the two stream treatments (low nutrient vs. high nutrient; Table S1 and Figure S4).

### 2.6 Relative telomere length measurement

A full description of the protocols for DNA preparation and measurement of relative telomere length is provided in the Appendix S1. In brief, telomere length was measured in all samples using the quantitative PCR (polymerase chain reaction) method described by Cawthon (2002). The universal Tel1b and Tel2b primers designed by Cawthon (2002) and modified by Epel et al. (2004) were used for amplification of the telomere repeats. The recombination activating gene 1 (RAG‐1) was chosen as the single‐copy gene and the *S*. *salar* RAG‐1 sequence (GenBank accession no.: JN132677) was used to design primers. The following forward and reverse RAG‐1 primers successfully amplified a single amplicon, as determined by melt curve analysis, and were subsequently used in the analysis:

SalmonRAG1‐F 5′‐TTGGAGGACCAATCCTCATTC‐3′ and
SalmonRAG1‐R 5′‐TCCGTGCATAGTTCCCATTC‐3′.

The telomere (T) and single‐copy gene (S) assays were performed on separate 96‐well plates, with each sample run in triplicate for each assay (see Appendix S1 for details of plate efficiencies and CV). qPCR data were then analysed using the qbase software for windows (Hellemans et al., 2007), following the same protocol as in McLennan et al. (2016).

### 2.7 Relative TERT expression measurement

To estimate telomerase expression, we designed a qPCR assay to measure the relative expression of the TERT gene, which is the catalytic subunit of the telomerase ribonucleoprotein complex (along with a telomerase RNA component). Full descriptions of the RNA/cDNA preparation and relative TERT expression measurement protocols are provided in the Appendix S1. The well‐established B paralogue of the elongation factor 1A gene (EF1A_B_) was used as the reference gene, and the same EF1A_B_ primers and probe outlined in Olsvik et al. (2005) were also used in this study. The Atlantic salmon TERT sequence (GenBank accession no.: XR_001320740) contains 16 exons and was used to design primers that spanned exon 6 (exon interval 88831916–88831755) and exon 7 (exon interval 88831622–88831521). The following forward and reverse TERT primers successfully amplified a single amplicon, as determined by melt curve analysis, and were subsequently used in the analysis:

SalmonTERT‐F 5′‐TGTCACATGTGCAGGAAGAG‐3′ and
SalmonTERT‐R 5′‐GAAGTCTGCCTGTCTGACAAA‐3′.

A single TERT amplicon was detected for each of the measured 175 individuals, providing evidence that the TERT gene was actively being expressed at the time of measurement. Amplification occurred relatively late in the thermal profile (with quantification cycles (Cqs) ranging from 27.27 to 31.43 out of a total of 45 cycles) suggesting that while measurable, TERT expression was potentially also relatively low; however, this would require further validation. The TERT and EF1A_B_ assays were performed on separate 96‐well plates, with each sample run in triplicate for each assay. Again, data were analysed using the qbase software.

### 2.8 Statistical methods

We ran a series of linear mixed effects models (lme4 and lmerTest functions in R version 3.5.1) (Bates et al., 2015; Kuznetsova et al., 2014) to examine whether *Relative telomere length* and *Relative TERT expression* varied as a function of nutrient treatment and the different habitat variables (Table 1). *Body Mass* and *Sex* were also included in the models because they have been previously shown to affect telomere dynamics in Atlantic salmon (Table 1; e.g. McLennan et al., 2016; McLennan et al., 2017). Habitat variables that differed both within a stream (i.e., per 5‐m subsection) and among streams (e.g. *Density*) were included in models as two separate explanatory predictors, following the within‐subject centring protocol outlined by van de Pol and Wright (2009). In doing so, each habitat variable was included as two independent predictors to evaluate broad‐scale among‐stream habitat effects (e.g., *mean_Density*) and finer scale within‐stream effects (e.g., *centered_Density*).

**TABLE 1:** Summary of the response and predictor variables used in the statistical analyses. See Section 2 for a more detailed description of each variable and an outline of each model. Linear mixed effect models also included Stream ID and Family ID as random effects to account for potential nonindependence between streams and/or siblings

Variable name	Variable description
*Relative telomere length*	Calculated for each individual as the ratio of telomere repeat copy number to a stable copy number gene (RAG‐1) using quantitative real‐time PCR. Log_10_‐transformed. Used as both response and continuous predictor variable
*Relative TERT expression*	Calculated for each individual as the ratio of TERT gene cDNA concentration to the concentration of a stably expressing reference gene (EF1A_B_) using quantitative real‐time PCR. Log_10_‐transformed. Response variable
*Treatment*	“High nutrient” treatment (analogue carcass pellet addition) or “low nutrient” treatment (without pellets). Five streams per treatment. Categorical predictor variable
*Sex*	Individuals were assigned as male or female using a conventional PCR sexing assay. Categorical predictor variable
*Body mass*	Somatic mass (±1.0 mg) of each individual measured upon recapture in the field when aged 1+ (i.e., ~15 months old). Continuous predictor variable
*Density*	Centered_Density was calculated as the number of 1+ salmon captured within each subsection of stream (5 m long) divided by the area (m^2^) of that subsection. Mean_Density was also calculated for each stream. Continuous predictor variable
*Flow Index*	Index of water velocity. Centered_Flow Index was calculated for each stream subsection, with values ranging from 1 (100% still water) to 6 (100% torrent). Mean_Flow Index was also calculated for each stream. Continuous predictor variable
*Depth*	Centered_Depth was calculated for each stream subsection based on the average of 10 depth measurements (m). Mean_Depth was also calculated for each stream. Continuous predictor variable
*Granulometric Index*	Index of substrate size. Centered_Granulometric Index was calculated for each stream subsection, with values ranging from 1 (100% sand) to 6 (100% bedrock surface). Mean_Granulometric Index was also calculated for each stream Continuous predictor variable

For the analysis of *Relative telomere length*, we established a core model that included three predictor variables: *Treatment* (whether the stream was high nutrient or low nutrient), *Body mass* and *Sex*. We then ran separate preliminary models that included the three core predictor variables and one of the four habitat variables of interest (based on *Density*, *Flow Index*, *Depth* or *Granulometric Index*) as two separate predicters (i.e., *Mean*_ and *Centered*_; see Table S2 for an overview of each preliminary model). Variance inflation factors (VIFs) were <2 for all predictor variables, indicating a lack of multicollinearity (Thompson et al., 2017). We also tested for an interaction between *Treatment* and each of the habitat variables. We then ran a final model (Table 2) that included the three core predictor variables and any habitat variables and/or interactions that were statistically significant (*p* < .05) in the preliminary models (which were the interactions *Treatment* × *Centered_Density* and *Treatment* × *Mean_Granulometric Index*). For this final model, we again calculated VIFs for each of the independent covariates (which were again less than 2 in all cases). The marginal *R* ^2^ value of this final model was calculated using the mumin package. Stream ID and Family ID were included as random effects in each mixed effect model to account for nonindependence between siblings and/or streams and their statistical significance was tested using the rand function.

**TABLE 2:** Statistical summary of the final linear mixed model explaining variation in relative telomere length (log_10_). Intercept represents females for the variable *Sex* and represents low nutrient streams for the variable *Treatment*. See Section 2 and Table S2 for an overview of the initial habitat variables considered. *Centered_Density* refers to within‐stream local salmon density, while *Mean_Granulometric Index* refers to differences among streams in substrate topography. *N* = 413

Fixed effects	Estimate	SE	df	*t*	*p*
Intercept	−2.203	.271	3.751	−8.125	.002
Treatment	1.954	.358	5.191	5.463	.002
Sex	0.004	.009	393.894	0.453	.650
Body mass	−0.008	.002	60.103	−4.514	<.001
Centered_Density	−0.135	.043	387.577	3.157	.002
Mean_Granulometric Index	0.568	.068	3.594	8.407	.002
Treatment × Centered_Density	0.119	.051	386.665	2.318	.021
Treatment × Mean_Granulometric Index	−0.485	.090	5.021	−5.382	.003
**Random effects**	**Variance**	**SD**			
Family ID	<0.001	0.025			
Stream ID	<0.001	0.007			
Residual	0.007	0.083			

We adopted the same approach for analysing *Relative TERT expression*, with four preliminary models (one per habitat feature) followed by a core model that also initially included *Relative telomere length* as a predictor variable to test whether there was a significant link between telomere length and TERT expression, although this was subsequently excluded from the core model (see Table S2 for an overview of each preliminary model). Interaction terms were not tested in these models, due to the smaller sample size. None of the preliminary models analysing variation in *Relative TERT expression* included significant terms (*p* < .05); therefore, a final model was not tested.

## 3 RESULTS

**FIGURE 1:**
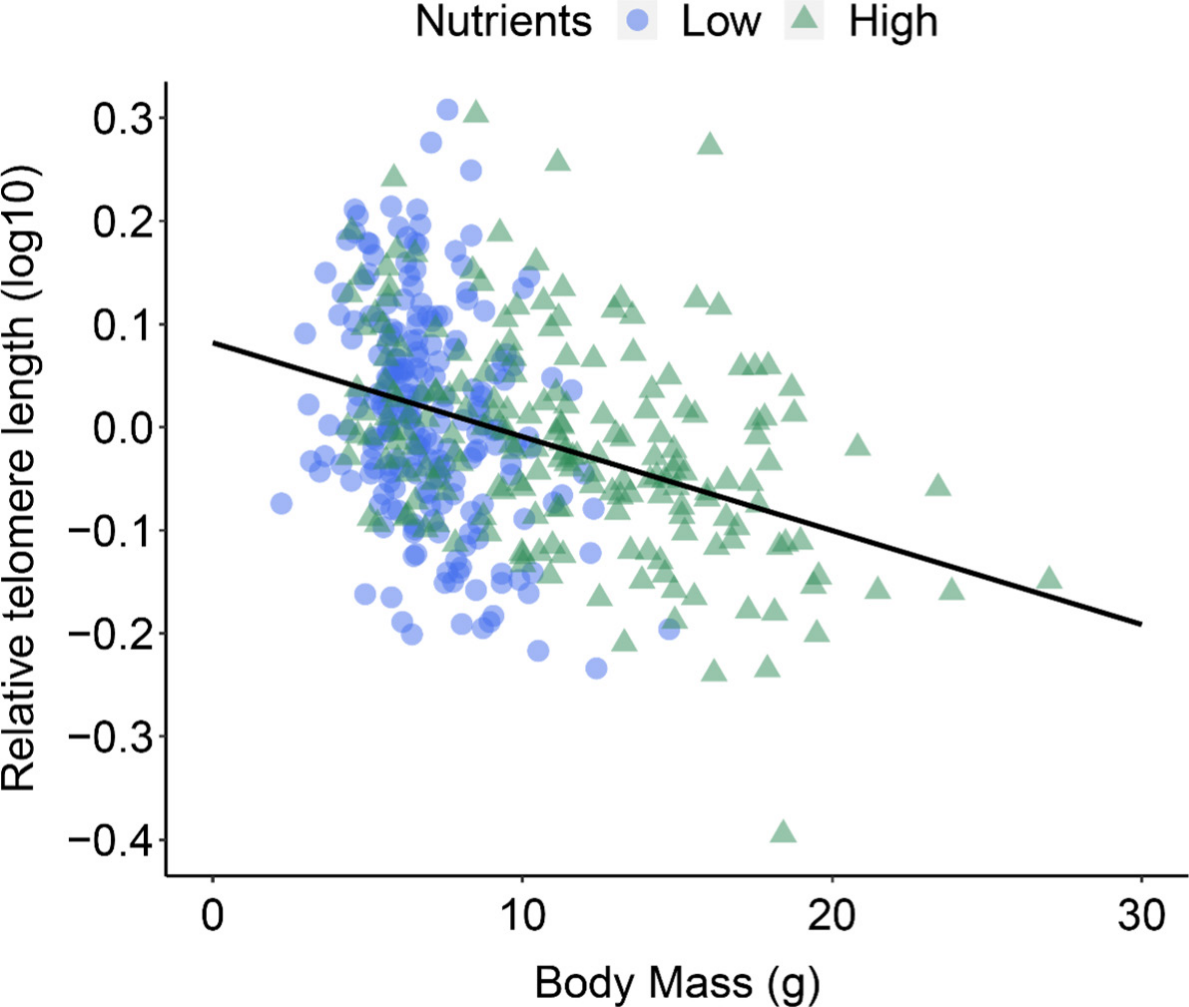
The relationship between the body mass (g) of focal juvenile salmon (aged 1+ years old) and their relative telomere lengths. Data plotted as individuals (*n* = 413). See Table 2

**FIGURE 2:**
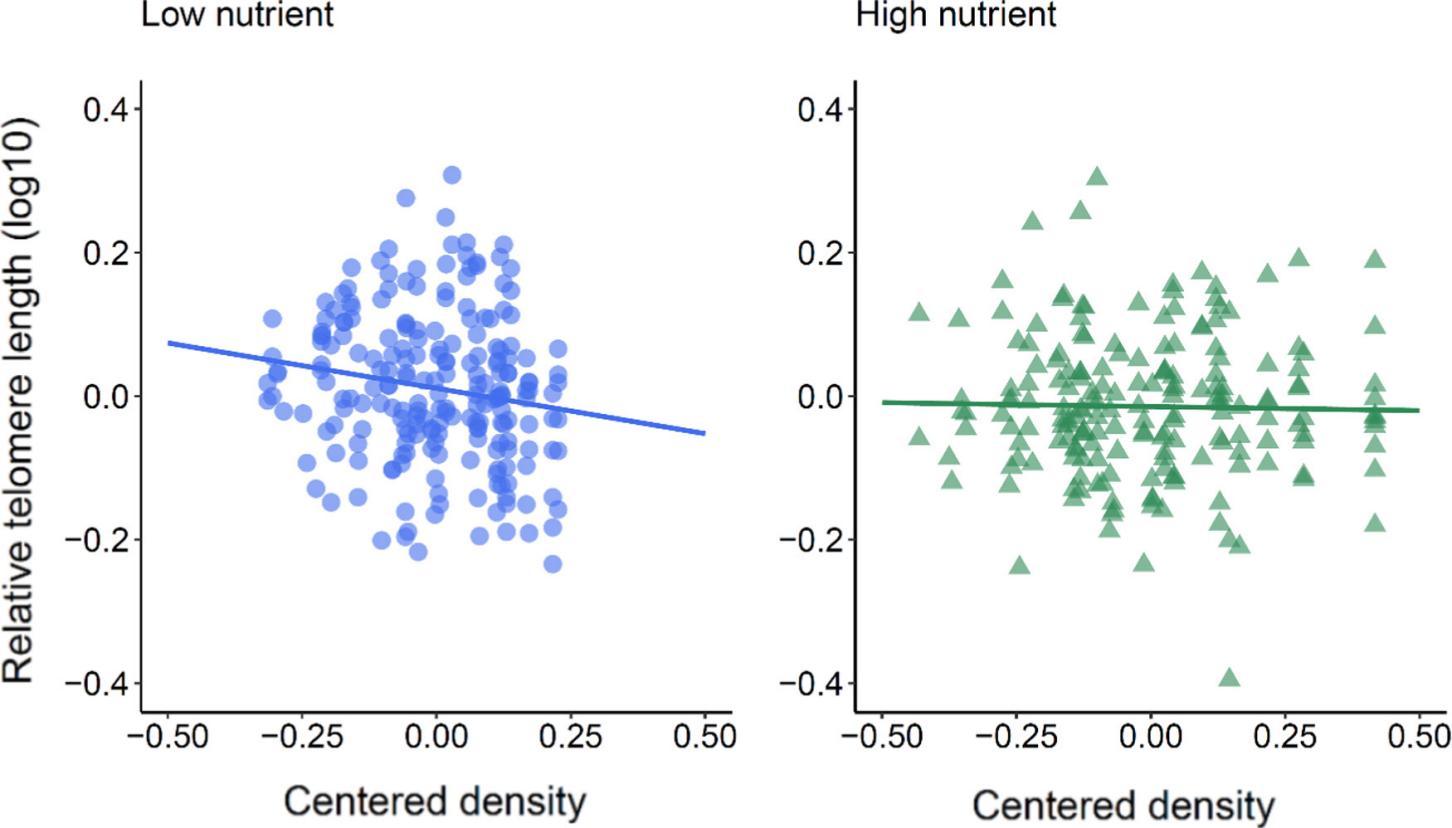
The relationship between the density of focal juvenile salmon (aged 1+ years old) in each 5‐m subsection of stream and the relative telomere length of those salmon. The centred density is a measure of local density correcting for variation in density among streams; see Section 2. Data plotted as individuals (*n* = 413). Blue circles represent individuals from low nutrient streams (*n* = 216), while green triangles represent individuals from the high nutrient streams (*n* = 197). See Table 2

**FIGURE 3:**
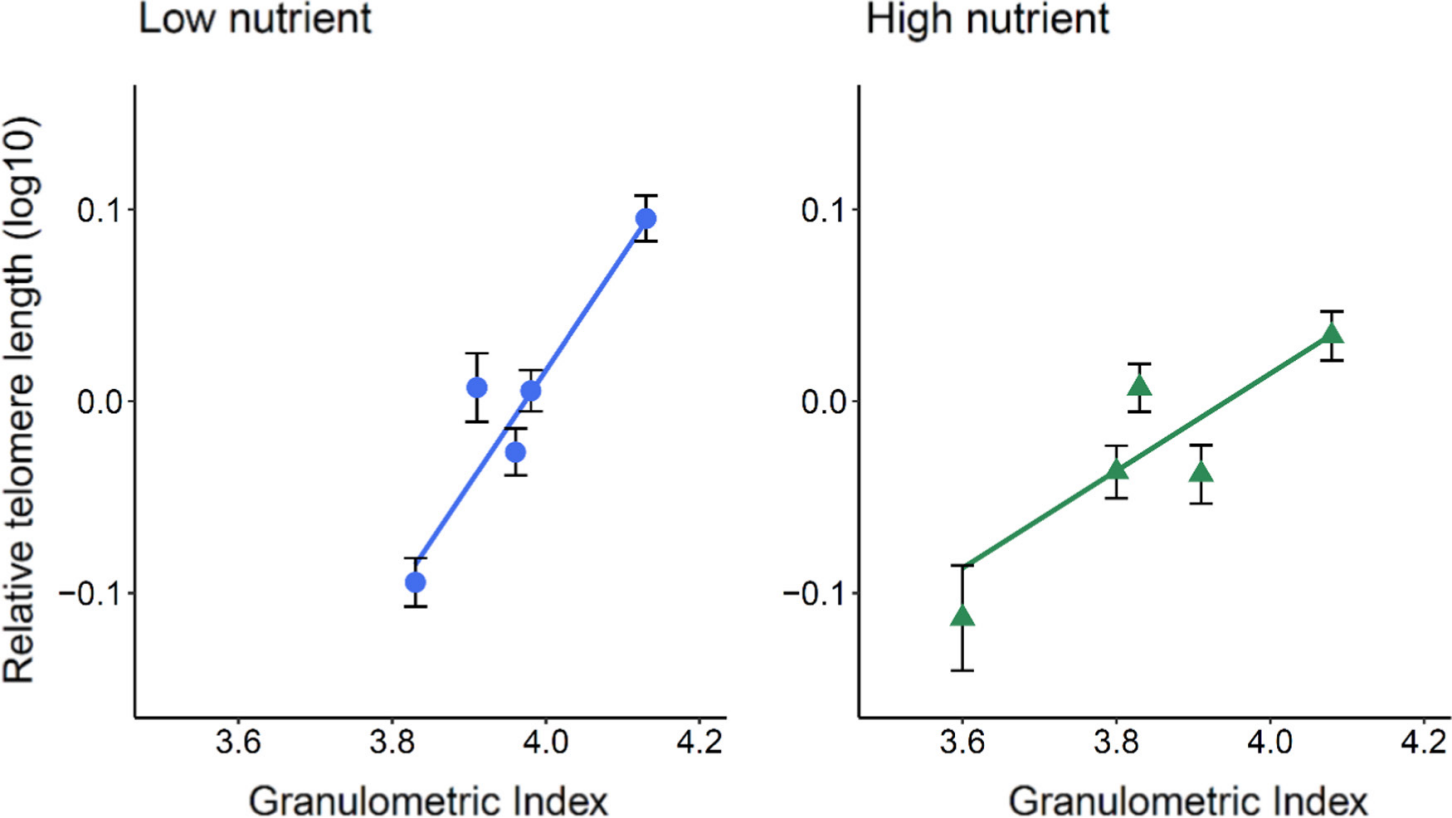
The relationship between a stream's mean granulometric index and the relative telomere length of the focal salmon (aged 1+ years old) captured within that stream. Data plotted as the mean of each stream ± 1SE, and regression line. Blue circles represent individuals from low nutrient streams (*n* = 216), while green triangles represent individuals from the high nutrient streams (*n* = 197). See Table 2

Relative telomere length was a negative function of body mass (*F*_1,60.1_ = 20.38, *p* < .001), with larger fish having relatively smaller telomeres (Table 2, Figure 1). Water depth and flow index (i.e., water velocity) were unrelated to relative telomere length, both within and among streams (Table S2). However, there was a significant within‐stream density by treatment effect (*F*_1,386.67_ = 5.38, *p* = .021, Table 2, Figure 2). Specifically, there was a significant negative relationship between salmon density and relative telomere length within the low nutrient streams, but this relationship was not apparent in the high nutrient streams, that is those streams that had undergone nutrient restoration (Table 3). There was also a significant granulometric index by treatment effect on relative telomere length among streams (*F*_1,5.02_ = 29.97, *p* = .002, Table 2, Figure 3); while salmon were more likely to have longer telomeres in streams dominated by cobbles and boulders (rather than finer particles such as pebbles and gravel) when reared in the low nutrient streams, we did not find evidence of a significant substrate effect in the high nutrient streams (Table 3). The marginal *R* ^2^ value for the final model was 0.31. There was a significant effect of family ($\chi_{1}^{2}$ = 9.59, *p* = .002), but not of stream ID ($\chi_{1}^{2}$ = 0.14, *p* > .05).

**TABLE 3:** Statistical summary of the linear mixed model testing whether the relationship between *Relative telomere length* and each of the final habitat variables significantly differed from 0; tested separately for each *Treatment*. *Centered_Density* refers to within‐stream local salmon density, while *Mean_Granulometric Index* refers to differences among streams in substrate topography. *N* = 413

Difference from zero: Fixed effects	Estimate	SE	df	*t*	*p*
Low nutrient					
Centered_Density	−0.135	.042	387.577	−3.157	.002
Mean_Granulometric Index	0.568	.068	3.594	8.407	.002
High nutrient					
Centered_Density	−0.016	.029	398.397	−0.561	.575
Mean_Granulometric Index	0.084	.064	10.346	1.299	.222

TERT gene expression was unrelated to relative telomere length (see Figure S5), nor was it significantly influenced by sex, body mass, nutrient treatment, or any of the four measured habitat variables—density, flow index, depth and granulometric index—at either the broader (among streams) or finer scale (within streams; Table S3).

## 4 DISCUSSION

Restoration of nutrients to human‐impacted oligotrophic streams is known to provide short‐term benefits for juvenile salmon, such as more invertebrate food and an associated faster rate of somatic growth (Auer et al., 2018; McLennan et al., 2019; Williams et al., 2009; Wipfli et al., 2003), but the longer term effects on the physiology of the salmon have been little tested. In this experiment, we show that addition of nutrients to oligotrophic streams mitigates the adverse effects on telomere dynamics of both higher local densities and a lower availability of cobble and boulder substrates. These results suggest that environmental pressures on the physiology of juvenile salmon are weakened when nutrients are restored, thereby reducing their impact on the rate of ageing.

In contrast, while the TERT gene appeared to be actively expressing at the time of measurement, the level of expression was unrelated to any of the measured habitat variables or to relative telomere length; this suggests that the level of TERT expression was not enough to maintain the telomere length of the juvenile salmon in poorer quality habitat. It also bears mentioning that while the main function of the TERT gene is to act as the catalytic subunit of telomerase (Srinivas et al., 2020), it may also serve other cellular functions, thereby adding a degree of noise to the relationship between TERT expression and telomerase activity. That considered, studies on telomere elongation mechanisms will benefit from future methodological advances in quantifying actual telomerase activity. We have previously shown that Atlantic salmon telomeres can increase in length at post‐embryonic juvenile stages (McLennan et al., 2018a) and potentially also at later adult life stages (McLennan et al., 2017); however, we did not quantify telomere repair mechanisms in either of those studies. In contrast to evidence from larger and/or longer‐lived endotherms, a number of ectotherm studies have found that telomere repair mechanisms (mostly by the enzyme telomerase) continue to be active at post‐embryonic life stages (Alibardi, 2015; Hatakeyama et al., 2016; Peterson et al., 2015; Yap et al., 2005; Yip et al., 2017). More specifically, studies on fish (Anchelin et al., 2011; Panasiak et al., 2020) and lizards (Ujvari et al., 2017) have shown that telomeres in ectotherms may lengthen and shorten at different specific phases of the life cycle. That being the case, it suggests that patterns of ectotherm telomere maintenance may depend more on life stage than being under the influence of environmental fluctuations, thus potentially explaining why telomerase activity was unrelated to any of the measured habitat variables in this study.

Relative telomere length was negatively correlated with body mass, with larger individuals having relatively smaller telomeres. Since all of the salmon were created by in vitro fertilization over a 3‐day period, and so were the same age when recaptured (again over a narrow time period), this variation in body size was caused by differing rates of growth rather than age‐specific differences. While fish had on average grown faster and so were larger in the high nutrient streams (McLennan et al., 2019), telomere lengths were not influenced by any interaction between the nutrient manipulation and growth rate, since telomere length decreased with increasing body size in a similar manner between treatments. Rate of growth has been linked to telomere dynamics among numerous different taxa (for a review see Monaghan & Ozanne, 2018). This has led to the hypothesis that telomere length (and rate of loss) may contribute to life history trade‐offs and growth rate optimization, as a faster rate of growth may be associated with an increased number of cell divisions and/or level of oxidative stress (Arendt, 2007; Kim et al., 2011; Smith et al., 2016), both of which accelerate the rate of telomere shortening. We have previously identified similar growth effects on juvenile salmon telomeres, both in the laboratory (McLennan et al., 2018b) and in the field (McLennan et al., 2016). Both of those previous studies focused on salmon juveniles that were only a few months old (age 0+), while in this current study the juveniles were 1 year older (age 1+). Combined, our studies suggest that attaining a larger body size may come at a cost of faster telomere attrition throughout the early freshwater life stages of Atlantic salmon. However, salmon are particularly unusual in that the majority of their adult body mass (up to 99%) is attained only once they have migrated out of their natal stream and into the ocean, even though this period may account for only one‐third of their lifespan. It has been reported that telomerase expression in fish is positively correlated with cell proliferation (Peterson et al., 2015; Yap et al., 2005) and active cell proliferation is still detectable in Atlantic salmon at least 6 months after transfer to the sea (Johnston et al., 2003). Therefore, while it is becoming clearer that negative growth effects on telomere length are at play during the earlier life stages, there may still be potential for this loss to be counterbalanced at a subsequent life stage.

In addition to the significant effect of growth rate, we also found that density had a significant within‐stream effect on telomere length when food was limited (i.e., in the low nutrient streams); fish in subsections with higher densities of focal salmon (aged 1+ years old) had shorter telomeres. However, this effect was not apparent in the high nutrient streams, where food abundance was roughly five times greater (McLennan et al., 2019). The negative relationship between density and telomere length observed within low nutrient streams is intriguing because higher densities can be stressful and lead to telomere shortening but, at the same time, are often indicative of better habitat quality. Juvenile salmon disperse from their streambed nest into adjacent microhabitats within a given section of stream (Einum et al., 2012). Microhabitat partitioning in freshwater fish populations can result from complex interactions between the relative quality of the individual microhabitats, as well as the competitive abilities and energy requirements of the individuals that are born among them (Auer et al., 2020; Kobler et al., 2011; Svanbäck & Bolnick, 2007; Wathen et al., 2019). This can then lead to more favourable microhabitats supporting a higher density of individuals and less favourable microhabitats supporting fewer, potentially less‐competitive conspecifics. Previous studies in other taxa have linked population density to a higher level of physiological stress (Montero et al., 1999; Trenzado et al., 2006) and a faster rate of telomere attrition (Kotrschal et al., 2007; Sohn et al., 2012). Alterations to the hypothalamic–pituitary–adrenal axis (such as an increase/decrease of glucocorticoid levels) have the potential to affect the oxidative balance within cells (e.g., via metabolic processes), thereby providing a mechanistic link between competitive pressure and an individual's rate of telomere loss (Angelier et al., 2018; Haussmann & Heidinger, 2015). We have also previously shown that early growth in field‐reared Atlantic salmon can be especially costly (in terms of telomere loss) at higher densities (McLennan et al., 2016). Therefore, while this may initially seem counterintuitive, in that higher densities of juvenile salmon should be expected to occur in microhabitats of higher quality, it may be that this reflects a trade‐off between habitat suitability and individual physiological state. In other words, better competing juvenile salmon that successfully gain access to better quality habitats may do so at a cost of faster telomere attrition.

The within‐stream density effect was only apparent in streams where nutrient levels were low. Since relative fish densities between the two treatments were similar but food levels were much lower in the low nutrient streams (McLennan et al., 2019), this indicates that levels of competition would have been higher. We have previously shown that, when the fish were 3 months old, microhabitat partitioning in the low nutrient streams was significantly influenced by their metabolic phenotype, with individuals of a higher family‐level metabolic rate occupying better quality habitats and potentially displacing lower metabolic rate individuals into less favourable microhabitats (Auer et al., 2020). No such effect was found in the high nutrient streams, probably because an individual's energy needs were met regardless of their positioning within a stream. This may partly explain the result from the present analysis, where effects of local salmon density on telomeres were only found if nutrient levels were low.

Focal salmon that had lived in the low nutrient streams were found to have longer telomeres if that stream was predominantly bedded with cobble‐sized substrate (sizes ranging from 64 to 256 mm). Unlike density, which we were able to partially standardize among streams by planting out identical numbers of eggs into each experimental stream, there was some variation among streams in substrate topography, with some having a greater density of cobbles. Cobble‐sized substrates are thought to increase habitat quality for Atlantic salmon juveniles aged 1+ (Heggenes et al., 1999; Jonsson & Jonsson, 2011) for several reasons. First, the visual barriers created by a scattering of cobbles reduce the rate of interactions among juvenile salmonids holding adjacent territories, so leading to reduced competition among fish (Dolinsek et al., 2007; Hasegawa & Yamamoto, 2009; Kalleberg, 1958; Reid et al., 2012; Venter et al., 2008). Second, the interstitial spaces that form between the cobbles provide suitably sized shelters. These interstitial refugia reduce the predation risk on a given fish, which in turn may be associated with a reduction in perceived predation pressure (Venter et al., 2008). Previous studies have identified links between predation pressure and telomere dynamics (Burraco et al., 2017; Kärkkäinen et al., 2019; Noguera & Velando, 2019; Olsson et al., 2010), but see also Monteforte et al. (2020). The interstitial refugia within the substratum also provide juvenile salmon with shelter from adverse stream conditions. These fish preferentially feed in faster flowing waters, since these often carry higher densities of drifting macroinvertebrate prey compared with slower flowing pools (Brooks et al., 2017; Nislow et al., 1998). However, the availability of sheltering spaces within the substrate provides hydraulic cover, allowing the conservation of energy when not actively feeding and/or when stream conditions become too unfavourable, for example during floods (Allouche, 2002). The latter is particularly true over winter, when the swimming capability of juvenile salmon decreases with water temperature (Johnson et al., 1996). Therefore, in the absence of suitable shelter, juvenile salmon may expend more energy than is otherwise necessary, which could then carry costs for the rate of telomere attrition, such as via an increased metabolic rate and an elevated production of ROS. The availability of sheltering space within the substratum can also reduce metabolism even when the fish is inactive (Millidine et al., 2006), possibly due to a reduction in stress, since cortisol levels are lower when shelter is accessible (Näslund et al., 2013). Combined, these studies support the idea that a lack of suitable shelter may carry costs for individual stress levels and hence the telomere dynamics of juvenile salmon.

Unlike the low nutrient streams, there was no evidence of a significant substrate‐size effect among the streams provided with nutrients. Due to the increase in food availability, the juvenile salmon in the high nutrient streams experienced a greater rate of growth and a larger body size at the time of sampling. It could be argued that a larger body size is advantageous in the avoidance of gape‐limited predators; however, the similar densities between our treatments would suggest that size‐structured predator–prey interactions were not significantly contributing to the overall population dynamics in this study. Perhaps more likely is that the reduced food availability led to a greater proportion of time spent foraging in the low nutrient streams. Studies have shown that a juvenile salmon's nutritional status (i.e., hunger level) is an important predictor of its foraging activity (Gotceitas & Godin, 1991; Vehanen, 2003). The relatively restricted feeding opportunities in the low nutrient streams may therefore have acted to strengthen the link between substrate size and juvenile salmon telomere length. In other words, fish in streams lacking both nutrients and cobbles will not only have had the stress of reduced shelter availability but may also have needed to invest more time and energy in foraging. Lastly, it might also be the case that we are witnessing an energetic trade‐off between locomotion (for the purposes of seeking out food) and somatic maintenance (Husak et al., 2016). That being the case, the five‐fold increase in food availability in the high nutrient streams (McLennan et al., 2019) may have reduced the swimming effort required to attain food, thereby allowing energy to be diverted into cellular maintenance processes such as oxidative damage repair and potentially weakening the link between substrate structure and telomere length.

We show here that habitat restoration of Scottish upland streams (in the form of nutrient addition) can mitigate the negative effects of other environmental factors on the telomere dynamics of the juvenile salmon that live within them. Whether this nutrient restoration may influence longer term senescence patterns remains to be seen, complicated further by the fact that telomere elongation mechanisms may come into play at a later stage in the life cycle. Irrespective, not only does this study highlight how human‐induced habitat degradation may act to strengthen negative environmental effects on telomere dynamics, it also demonstrates that restoration of these impacted habitats may counteract such effects. That being the case, measurements of telomere dynamics may prove a useful means to assess habitat suitability and to evaluate the success of conservation management in the future.

## AUTHOR CONTRIBUTIONS

D.M., S.K.A., S.M. and N.B.M. conceived the ideas and designed the methodology; D.M., S.K.A., S.M., L.M., G.J.A., W.B., J.D. and N.B.M collected the data; D.M., S.K.A. and N.B.M analysed the data and led the writing of the manuscript. All authors contributed critically to the drafts and gave final approval for publication.

## CONFLICT OF INTEREST

We declare no conflict of interest.

## Supporting information

Supplementary Material

## Data Availability

Data are available via the Dryad Digital Repository https://doi.org/10.5061/dryad.2v6wwpzn6.
