# Supplementary material for: Habitat restoration weakens negative environmental effects on telomere dynamics
Source: Mol Ecol. 2021 Jul 22;31(23):6100–13. doi: 10.1111/mec.15980 (PMC10286771; doi:10.1111/mec.15980)
Supplement: Supplementary file 1 — Supplementary Material [file MEC-31-6100-s001.docx]

**
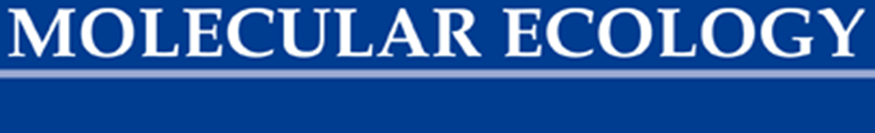
**

**Supplemental Information for:**

**Habitat restoration weakens negative environmental effects on telomere dynamics**

Darryl McLennan^1^, Sonya K. Auer^1,2^, Simon McKelvey^3^, Lynn McKelvey^3^, Graeme Anderson^1^, Winnie Boner^1^, Jessica Duprez^1^, and Neil B. Metcalfe^1^

^1^ Institute of Biodiversity, Animal Health and Comparative Medicine, Graham Kerr building, University of Glasgow, Glasgow, G12 8QQ UK.

^2^ Department of Biology, Williams College, Williamstown, MA 01267 USA

^3^ Cromarty Firth Fishery Trust, Inverness, IV2 3HF UK.

Corresponding author: [darrylmclennan@outlook.com](mailto:darrylmclennan@outlook.com)

**S1 | Parental assignment and sexing of the recaptured focal juvenile salmon**

The recaptured focal salmon (i.e., those that were planted out in March 2016 and were 1+ years old at the time of recapture) were genotyped and assigned to one of the 30 focal families by commercial suppliers (Landcatch Natural Selection Ltd, Stirling, Scotland). DNA was extracted from the parental fin clip tissue samples using an E-Z 96 tissue DNA Tissue kit (Omega Bio-Tek, Georgia, USA), while DNA was extracted from the offspring fin clip tissue samples using a DNeasy Blood and Tissue Kit (Qiagen) as part of the DNA preparation for the telomere analysis (see section S2 below). Fish were then genotyped using a panel of 110 informative SNP markers scattered across the genome using individual end point PCR assays (KASP TM technology, UK). Parentage assignment by exclusion was carried out blind to experimental treatments with the programme Vitassign 8.3 (Vandeputte, Mauger & Dupont-Nivet 2006) with some modifications to allow the analysis of more than 100 markers. ~90% of all captured fish were successfully assigned to one of our 30 focal families, with the other 10% presumably being immigrating fish from other stocked non-experimental streams that were part of the same catchment. All salmon offspring were also sexed by the same commercial supplier and using the same DNA sample as for the parental assignment. In brief, sex was assigned using a sex determination qPCR assay. Primer and probe sequences were based on the *Oncorhynchus mykiss* sequences outlined in Yano *et al.* (2012), but with Atlantic salmon specific modifications. Presence or absence of an amplicon from the sexually dimorphic on the Y-chromosome gene (sdY) was used to determine whether individuals were genetically male or female, using a FAM-labelled Taqman probe. The assay also included a co-amplified endogenous control, targeting the housekeeping gene Elongation Factor-alpha (ELF-α), using a HEX labelled Taqman probe. Therefore, individuals that resulted in the amplification of both PCR products were identified as male, while individuals that only resulted in the amplification of the ELF-α PCR product were identified as female.

**S2 | Relative telomere length measurement**

DNA was extracted from the adipose fin tissue of each focal fish using the DNeasy Blood and Tissue Kit (Qiagen), following the manufacturer’s protocol. The fin tissue was lysed in 180 µl buffer ATL + 20 µl of proteinase K solution (20 mg/ml). Each set of DNA extractions conducted also included a negative control that contained all the reagents, but without any tissue. DNA concentration and purity were measured spectrophotometrically using a Nanodrop 8000, which confirmed that all samples met the recommended A260/280 ratio and had a DNA concentration > 20 ng/µl.

Relative telomere length was measured in all samples using the quantitative PCR method described by Cawthon (2002). The universal Tel1b and Tel2b primers designed by Cawthon (2002) and modified by Epel *et al.* (2004) were used for amplification of the telomere repeats. The recombination activating gene 1 (RAG-1) was chosen as the single copy gene and the *Salmo salar* RAG-1 sequence (Genbank accession number: JN132677) was used to design primers. The following forward and reverse RAG-1 primers successfully amplified a single amplicon, as determined by melt curve analysis, and were subsequently used in the analysis:

SalmonRAG1-F 5’- TTG GAG GAC CAA TCC TCA TTC -3’ and

SalmonRAG1-R 5’- TCC GTG CAT AGT TCC CAT TC -3’.

The telomere assay thermal profile was 15 min at 95°C followed by 27 cycles of 15 secs at 95°C, 30 secs at 58°C and 30 secs at 72°C. This was followed by a melt curve analysis. The thermal profile of the RAG-1 assay was 15 min at 95°C followed by 40 cycles of 15 secs at 95°C, 30 secs at 60°C and 30 secs at 72°C. Again, this was followed by a melt curve profile. PCRs were performed on an Mx3005P qPCR system (Agilent). The telomere (T) and single copy gene (S) assays were performed on separate 96 well plates, with each sample run in triplicate for each assay. In addition to the samples, each plate also included a six-fold serial dilution of a reference sample (0.625 - 20 ng/well), and a non-target control (NTC). The DNA for the serial dilution was a pool of DNA from 200 individuals, with 20 individuals drawn from each of the 10 experimental streams. The NTCs contained all reaction components apart from DNA and were included on each plate (in triplicate) to check for non-specific binding and potential contamination between sample wells.

Each reaction contained 12.5µl 2x ABsolute Blue qPCR SYBR Green Mix low ROX (Fisher Scientific), forward and reverse primers and DNA (wells containing sample, standard) or water (wells containing NTC) in a total volume of 25µl. Both T and S assays were performed using 5ng of DNA/well. Primer concentrations were 500nM for the telomere assay (Tel1b and Tel2b) and 200nM for the RAG-1 assay (SalmonRAG1-F and SalmonRAG1-R). Samples were randomly distributed across 30 sets of PCR plates (T and S assays). qPCR data were analysed using the qBASE software for windows (Hellemans *et al.* 2007), as described in McLennan *et al.* (2016). The mean efficiencies of the telomere and RAG-1 assays were 108.50 and 93.31 respectively, with the qBASE software additionally helping to control for differences in amplification efficiency between targets and plates (assessed from the standard curve of each plate). The qBASE software also helped to correct for inter-run variation by using three points from the standard curve (2.5, 5 and 10 ng/well) as inter-run calibrators. The standard curve was also used to calculate an inter-assay coefficient of variation of the T/S ratios (which was 8.85). Out of the 433 focal fish captured in the field, telomere length was successfully measured for 413 individuals. The other 20 individuals were excluded either because of very low DNA concentrations (< 20 ng/µl) due to very small fin clip tissue samples, or because the sample lay out with the acceptable limits of the RAG-1 single copy gene standard curve. All outlier samples were repeated once (from the DNA dilution step onwards) before a decision on exclusion was made.

**S3 | TERT expression measurement**

Prior to RNA extraction, the muscle tissue was removed from the Allprotect reagent, any residual reagent was removed, and the muscle tissue was weighed (to 0.001 g). The tissue was disrupted in a 2 ml microcentrifuge tube that contained a 5 mm stainless steel bead, using the TissueLyser homogenizing system. Each homogenate was diluted in Buffer RLT (Qiagen) to contain 12.5 mg of tissue in a total volume of 400 µl. The diluted homogenates were then centrifuged and 300 µl of the supernatant was used in the subsequent RNA extraction. RNA was extracted from the muscle tissue using the RNeasy Blood and Tissue kit (Qiagen), following the manufacturer’s protocol and including the additional DNase digestion step using the RNase-Free DNase Set (Qiagen). RNA quality and quantity were checked using the 4200 Tapestation System (Agilent), with a mean RNA Integrity Number (RIN) of 9.8 across all samples. Reverse transcription reactions (containing 60 ng/µl RNA) were performed to convert RNA to cDNA using the QuantiTect Reverse Transcription Kit (Qiagen), following the manufacturer’s protocol.

Expression of the TERT gene was analysed by qPCR. The Atlantic salmon TERT sequence (Genbank accession number: XR_001320740) contains 16 exons and was used to design primers that spanned exon 6 (exon interval 88831916 - 88831755) and exon 7 (exon interval 88831622 - 88831521). The following forward and reverse TERT primers successfully amplified a single amplicon, as determined by melt curve analysis, and were subsequently used in the analysis:

SalmonTERT-F 5’- TGT CAC ATG TGC AGG AAG AG -3’ and

SalmonTERT-R 5’- GAA GTC TGC CTG TCT GAC AAA -3’.

For the reference gene, the well-established B paralog of the elongation factor 1A gene (EF1A_B_) was used. Olsvik *et al.* (2005) found this to be one of the most stable reference genes in Atlantic salmon (including muscle tissue) and the same EF1A_B_ primers and probe suggested by Olsvik *et al.* (2005) were also used in this study. The TERT thermal profile was 15 min at 95°C followed by 45 cycles of 15 secs at 95°C, 30 secs at 60°C and 30 secs at 72°C. This was followed by a melt curve analysis. The EF1A_B_ thermal profile was 3 min at 95°C followed by 50 cycles of 15 secs at 95°C and 1 min at 60°C. PCRs were performed on an Mx3005P qPCR system (Agilent). The TERT and EF1A_B_ assays were performed on separate 96 well plates, with each sample run in triplicate for each assay. In addition to the samples, each plate also included a six-fold serial dilution of a reference sample (5 - 160 ng/well for the TERT assay and 2.5 – 80 ng/well for the EF1A_B_ assay), and a non-target control (NTC). The cDNA used for the serial dilution was from a pool of RNA drawn from 50 of the 175 focal individuals. The NTCs contained all reaction components apart from cDNA and were included on each plate (in triplicate) to check for non-specific binding and potential contamination between sample wells.

For the TERT assay, each reaction contained 12.5µl 2x ABsolute Blue qPCR SYBR Green Mix low ROX (Fisher Scientific), forward and reverse primers (200nM each) and cDNA (wells containing sample (40 ng) or standard 5 – 160 ng)) or water (wells containing NTC) in a total volume of 25µl. For the EF1A_B_ assay, each reaction contained 10 µl of Brilliant III Ultra-Fast qPCR Master Mix (Agilent Technologies), reference dye (ROX 30 nM), forward and reverse primers (400 nM), probe (200nM) and cDNA (wells containing sample (20 ng) or standard 2.5 – 80 ng)) or water (wells containing NTC) in a total volume of 20µl. Samples were randomly distributed across eight sets of PCR plates. Again, data were analysed using the qBASE software. The mean efficiencies of the TERT and EF1A_B_ assays were 105.9 and 107.9 respectively. Three points from the standard curve (5, 20 and 80 ng/well) were used as inter-run calibrators during the qBASE analysis. The standard curve was also used to calculate an inter-assay coefficient of variability of the TERT expression data (which was 9.76). TERT expression was successfully measured in all 175 fish that were sampled for muscle tissue in the field. 24 of the RNA samples from our experimental fish (pre – reverse transcription) were randomly selected and run on the TERT assay, to provide evidence that genomic DNA contamination was not a confounding factor in our sample set.

**Table S1. Summary of the four measured habitat variables.** Means and standard errors are given for both the low nutrient streams (n = 5) and the high nutrient streams (n = 5). Linear mixed effect models tested for potential habitat differences between the two stream treatments. Stream ID was included as a random effect to control for non-independence of subsections within the same stream. See Fig. S4.

|  | Low parental  nutrient | | High parental  nutrient | | Difference between treatments | | | |
| --- | --- | --- | --- | --- | --- | --- | --- | --- |
|  |  |  |  |  |  |  |  |  |
|  | Mean | SE | Mean | SE | NUM DF | DEN DF | F | p |
| *Density (1+ salmon per m^2^)* | 0.361 | 0.191 | 0.357 | 0.282 | 1 | 7.672 | 0.003 | 0.960 |
| *Flow Index* | 4.071 | 0.078 | 3.759 | 0.081 | 1 | 8.136 | 2.251 | 0.171 |
| *Depth (m)* | 0.117 | 0.004 | 0.117 | 0.007 | 1 | 7.039 | <0.001 | 0.994 |
| *Granulometric Index* | 3.989 | 0.0278 | 3.841 | 0.037 | 1 | 8.007 | 2.403 | 0.160 |

**Table S2:** **Statistical summary of the four preliminary linear mixed models testing for habitat effects on *Relative telomere length* (log 10).** Each habitat variable was tested in a separate model, along with the three core predictor variables (*Treatment*, *Sex* and *Body Mass*). Each habitat variable was included in the model as two separate predictors, in order to assess both among-stream (e.g. *mean_*) and within-stream (e.g. *centered_*) effects (see materials and methods for details). Stream ID and Family ID were included as random effects in each model to account for potential non-independence between streams and/or siblings. Significant variables (Type III p < 0.05) were then included in the final model (see Table 2). N = 413 for each model.

| **Model**  **#** | **Predictor** | **Num**  **DF** | **Den**  **DF** | **F** | **p** |
| --- | --- | --- | --- | --- | --- |
| Core | Treatment | 1 | 32.09 | 0.0402 | 0.8424 |
| model | Sex | 1 | 393.51 | 0.2746 | 0.6006 |
|  | Body Mass | 1 | 313.76 | 13.4132 | 0.0003 |
|  | Treatment * Body Mass | 1 | 335.74 | 0.5076 | 0.4767 |
|  |  |  |  |  |  |
| 1 | Treatment | 1 | 6.53 | 0.3345 | 0.5824 |
|  | Sex | 1 | 391.62 | 0.2322 | 0.6301 |
|  | Body Mass | 1 | 292.84 | 11.4614 | 0.0008 |
|  | Centered_Density | 1 | 394.68 | 8.6932 | 0.0034 |
|  | Mean_Density | 1 | 6.55 | 2.2112 | 0.1834 |
|  | Treatment * Centered_Density | 1 | 386.17 | 4.8252 | 0.0286 |
|  | Treatment * Mean_Density | 1 | 6.26 | 0.3143 | 0.5945 |
|  |  |  |  |  |  |
| 2 | Treatment | 1 | 5.84 | 0.0311 | 0.8661 |
|  | Sex | 1 | 391.34 | 0.1310 | 0.7176 |
|  | Body Mass | 1 | 326.55 | 11.1694 | 0.0009 |
|  | Centered_Flow Index | 1 | 394.11 | 0.0000 | 0.9975 |
|  | Mean_Flow Index | 1 | 6.03 | 0.2057 | 0.6660 |
|  | Treatment * Centered_Flow Index | 1 | 394.16 | 2.7017 | 0.1010 |
|  | Treatment * Mean_Flow Index | 1 | 5.93 | 0.0292 | 0.8701 |
|  |  |  |  |  |  |
| 3 | Treatment | 1 | 5.91 | 0.8201 | 0.4005 |
|  | Sex | 1 | 393.67 | 0.1719 | 0.6787 |
|  | Body Mass | 1 | 252.32 | 12.6886 | 0.0004 |
|  | Centered_Depth | 1 | 396.43 | 1.1738 | 0.2793 |
|  | Mean_Depth | 1 | 6.00 | 1.7631 | 0.2325 |
|  | Treatment * Centered_Depth | 1 | 391.74 | 2.0630 | 0.1517 |
|  | Treatment * Mean_Depth | 1 | 5.92 | 0.8566 | 0.3908 |
|  |  |  |  |  |  |
| 4 | Treatment | 1 | 5.02 | 30.2094 | 0.0027 |
|  | Sex | 1 | 396.59 | 0.0678 | 0.7946 |
|  | Body Mass | 1 | 57.17 | 20.9088 | <0.0001 |
|  | Centered_Granulometric Index | 1 | 389.96 | 0.4886 | 0.4850 |
|  | Mean_Granulometric Index | 1 | 5.89 | 44.2271 | 0.0006 |
|  | Treatment * Centered_Granulometric Index | 1 | 390.58 | 2.9412 | 0.0871 |
|  | Treatment * Mean_Granulometric Index | 1 | 4.85 | 29.3502 | 0.0032 |

**Table S3: Statistical summary of the four preliminary linear mixed models testing for habitat effects on *Relative* *TERT expression* (log 10).** Each habitat variable was tested in a separate model, along with the three core predictor variables (*Treatment*, *Sex* and *Body Mass*). *Relative telomere length* was initially included in the core model to test whether it was significantly linked to TERT expression but was then subsequently excluded from the core model. Each habitat variable was included in the model as two separate predictors, in order to assess both among-stream (e.g. *mean_*) and within-stream (e.g. *centered_*) effects (see materials and methods for details). Stream ID and Family ID were included as random effects in each model to account for potential non-independence between streams and/or siblings. None of models included significant terms (Type III p < 0.05); therefore, a final model was not tested. N = 175 for each model.

| **Model #** | **Predictor** | **Num DF** | **Den DF** | **F** | **p** |
| --- | --- | --- | --- | --- | --- |
| Core | Treatment | 1 | 9.804 | 0.3015 | 0.5952 |
|  | Sex | 1 | 158.630 | 0.0332 | 0.8556 |
|  | Body Mass | 1 | 131.796 | 0.6247 | 0.4307 |
|  | Relative telomere length (log10) | 1 | 161.218 | 0.0003 | 0.9869 |
|  |  |  |  |  |  |
| 5 | Treatment | 1 | 9.299 | 0.0954 | 0.7642 |
|  | Sex | 1 | 163.273 | 0.0748 | 0.7848 |
|  | Body Mass | 1 | 146.499 | 1.5261 | 0.2187 |
|  | Centered_Density | 1 | 165.727 | 0.0932 | 0.7606 |
|  | Mean_Density | 1 | 7.785 | 1.7729 | 0.2207 |
|  |  |  |  |  |  |
| 6 | Treatment | 1 | 8.790 | 0.3511 | 0.5684 |
|  | Sex | 1 | 162.830 | 0.0700 | 0.7916 |
|  | Body Mass | 1 | 136.741 | 1.0365 | 0.3104 |
|  | Centered_Flow Index | 1 | 164.240 | 0.4819 | 0.4886 |
|  | Mean_Flow Index | 1 | 7.161 | 0.0972 | 0.7641 |
|  |  |  |  |  |  |
| 7 | Treatment | 1 | 8.837 | 0.1810 | 0.6807 |
|  | Sex | 1 | 162.741 | 0.0901 | 0.7644 |
|  | Body Mass | 1 | 139.545 | 1.3428 | 0.2485 |
|  | Centered_Depth | 1 | 161.918 | 1.4012 | 0.2383 |
|  | Mean_Depth | 1 | 7.214 | 0.3969 | 0.5481 |
|  |  |  |  |  |  |
| 8 | Treatment | 1 | 7.728 | 0.7214 | 0.4212 |
|  | Sex | 1 | 162.098 | 0.1019 | 0.7500 |
|  | Body Mass | 1 | 167.630 | 1.6971 | 0.1945 |
|  | Centered_Granulometric Index | 1 | 161.660 | 0.5431 | 0.4622 |
|  | Mean_Granulometric Index | 1 | 8.654 | 1.0632 | 0.3304 |


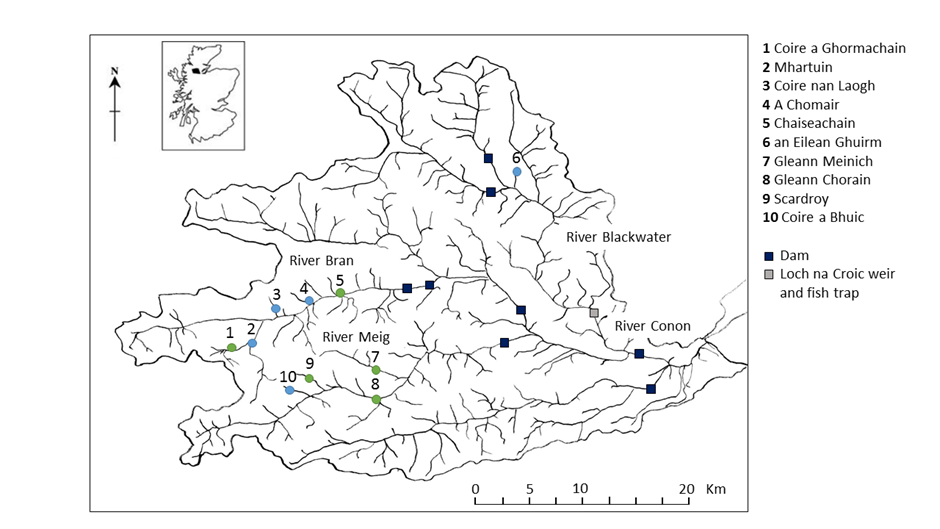


**Fig. S1.** Map of the River Conon catchment in Northern Scotland, including the location of study streams (green represents high nutrient streams, while blue represents low nutrient streams), key hydroelectric dams, and trap for collecting returning adult salmon on their return spawning migration from the sea. Figure taken from Auer *et al.* (2018).

**
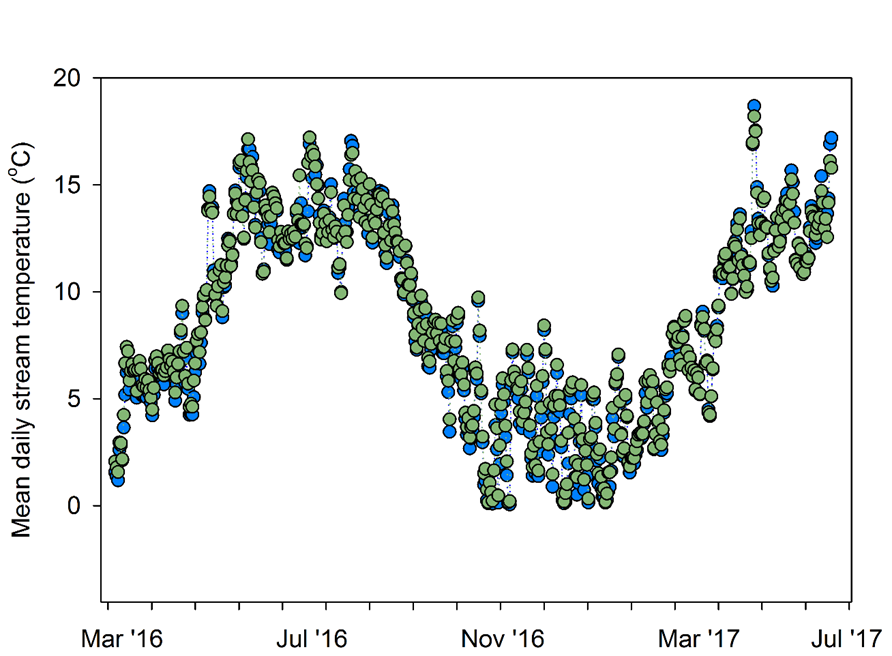
**

**Fig. S2.** Temporal changes in daily water temperature in 8 of the study streams. Green represents high nutrient streams (n = 4), while blue represents low nutrient streams (n = 4). Temperature was measured from the time that the focal eggs were planted out to the recapture of the focal juvenile salmon at age 1+ years old (March 2016 – July 2017). Figure taken from McLennan *et al.* (2019).


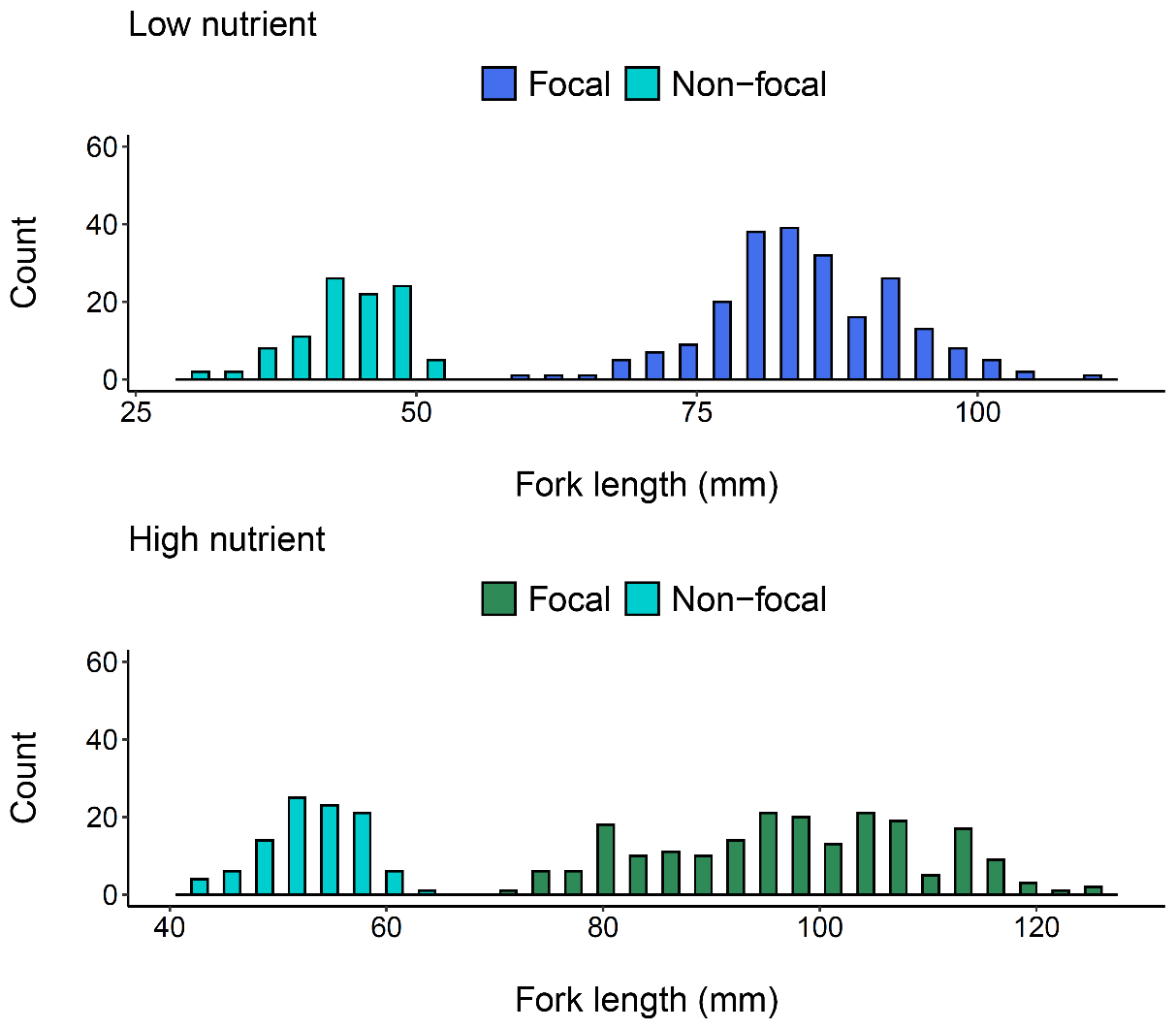


**Fig. S3.** Difference in distribution of fork length (mm) between the focal cohort of juvenile salmon (those that were planted out in March 2016 and were 1+ years old at the time of recapture, n=433) and the non-focal cohort of salmon (those that were planted out the following year in March 2017 and were 0+ years old at the time of recapture, n=200). Note the difference in scale on the x axis between the low and high nutrient samples.


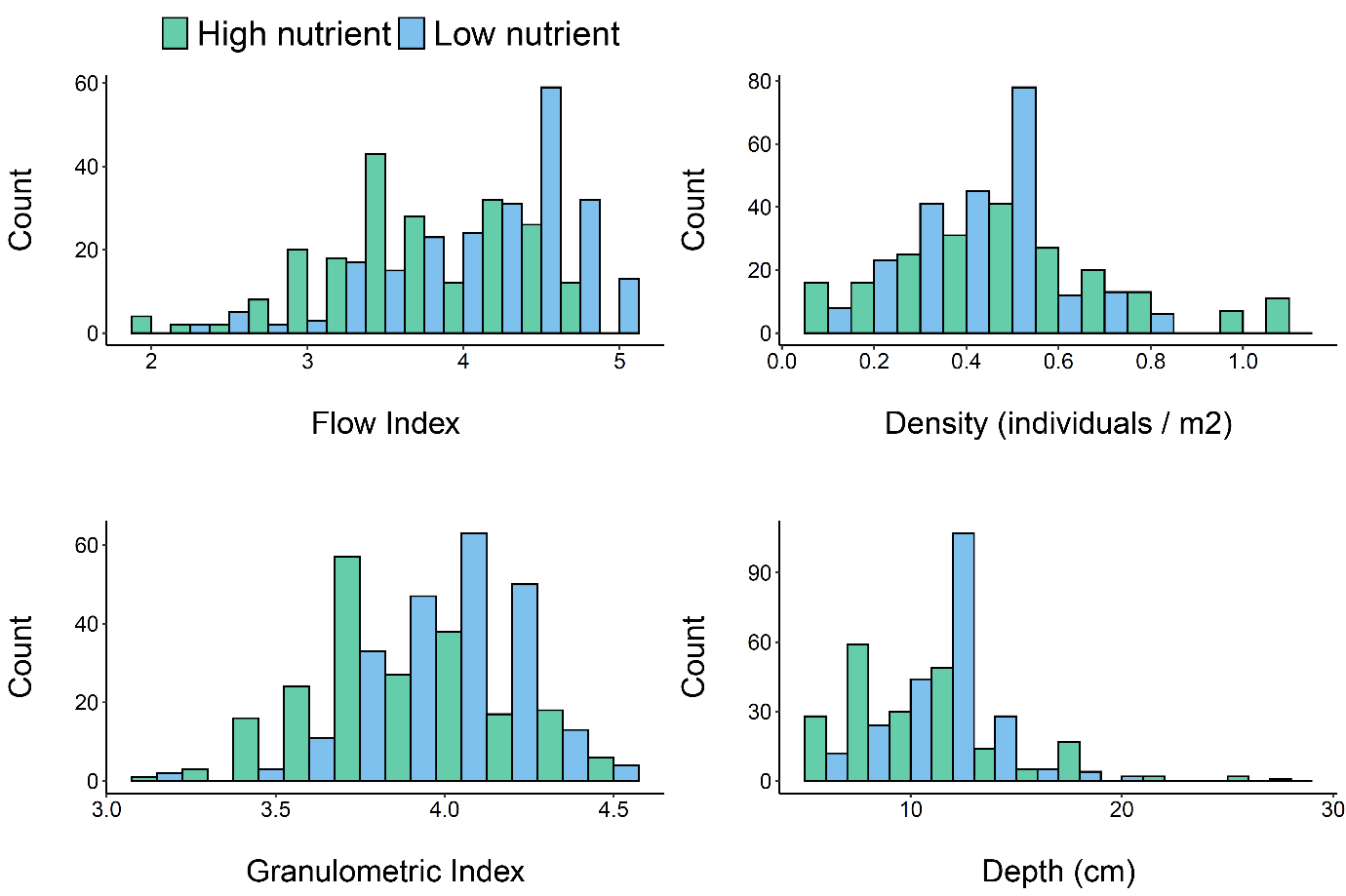


**Fig. S4.** Frequency distribution of the four measured habitat variables. Green represents high nutrient streams (n = 5), while blue represents low nutrient streams (n = 5). See Table S1.

**
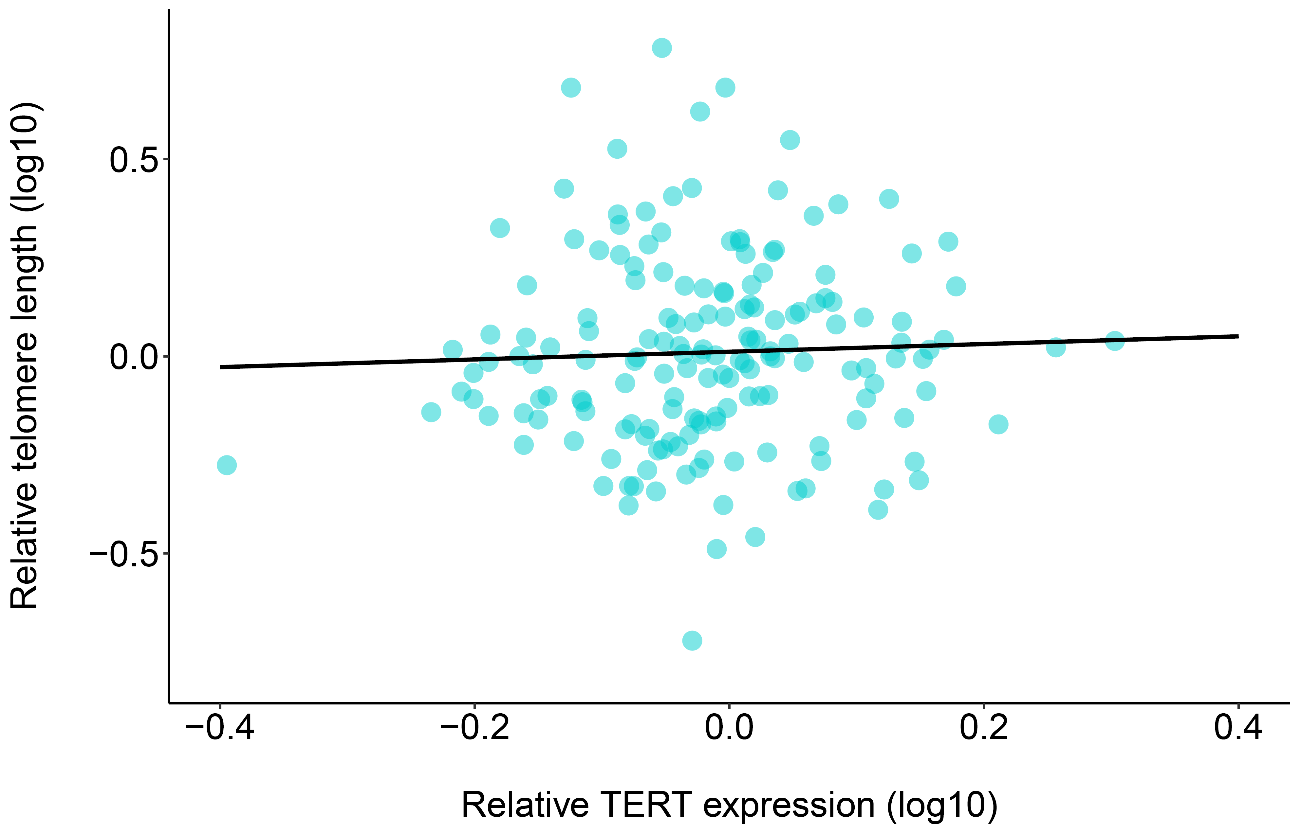
**

**Fig S5.** The non-significant relationship between TERT gene expression and the relative telomere length of the focal salmon (aged 1+ years). Data plotted as individuals (n=175). See Table S3.

**References**

Auer, S.K., Anderson, G.J., McKelvey, S., Bassar, R.D., McLennan, D., Armstrong, J.D., Nislow, K.H., Downie, H.K., McKelvey, L., Morgan, T.A.J., Salin, K., Orrell, D.L., Gauthey, L., Reid, T.C. & Metcalfe, N.B. (2018) Nutrients from salmon parents alter selection pressures on their offspring. *Ecology Letters,* **21,** 287-295.

Cawthon, R.M. (2002) Telomere measurement by quantitative PCR. *Nucleic Acids Research,* **30**.

Epel, E.S., Blackburn, E.H., Lin, J., Dhabhar, F.S., Adler, N.E., Morrow, J.D. & Cawthon, R.M. (2004) Accelerated telomere shortening in response to life stress. *Proceedings of the National Academy of Sciences of the United States of America,* **101,** 17312-17315.

Hellemans, J., Mortier, G., De Paepe, A., Speleman, F. & Vandesompele, J. (2007) qBase relative quantification framework and software for management and automated analysis of real-time quantitative PCR data. *Genome biology,* **8,** R19.

McLennan, D., Armstrong, J.D., Stewart, D.C., Mckelvey, S., Boner, W., Monaghan, P. & Metcalfe, N.B. (2016) Interactions between parental traits, environmental harshness and growth rate in determining telomere length in wild juvenile salmon. *Molecular Ecology,* **25,** 5425-5438.

McLennan, D., Auer, S.K., Anderson, G.J., Reid, T.C., Bassar, R.D., Stewart, D.C., Cauwelier, E., Sampayo, J., McKelvey, S., Nislow, K.H., Armstrong, J.D. & Metcalfe, N.B. (2019) Simulating nutrient release from parental carcasses increases the growth, biomass and genetic diversity of juvenile Atlantic salmon. *Journal of Applied Ecology,* **56,** 1937-1947.

Olsvik, P.A., Lie, K.K., Jordal, A.-E.O., Nilsen, T.O. & Hordvik, I. (2005) Evaluation of potential reference genes in real-time RT-PCR studies of Atlantic salmon. *BMC Molecular Biology,* **6,** 1-9.

Vandeputte, M., Mauger, S. & Dupont-Nivet, M. (2006) An evaluation of allowing for mismatches as a way to manage genotyping errors in parentage assignment by exclusion. *Molecular Ecology Notes,* **6,** 265-267.

Yano, A., Guyomard, R., Nicol, B., Jouanno, E., Quillet, E., Klopp, C., Cabau, C., Bouchez, O., Fostier, A. & Guiguen, Y. (2012) An immune-related gene evolved into the master sex-determining gene in rainbow trout, *Oncorhynchus mykiss*. *Current Biology,* **22,** 1423-1428.
